# Evolving Power Dynamics in Global Health: From Biomedical Hegemony to Market Dynamics in Global Health Financing; A Response to the Recent Commentaries

**DOI:** 10.34172/ijhpm.2023.8264

**Published:** 2023-10-02

**Authors:** Samuel Lassa, Muhammed Saddiq, Jenny Owen, Christopher Burton, Julie Balen

**Affiliations:** ^1^School of Health and Health Related Research, University of Sheffield, Sheffield, UK; ^2^Division of Population Health, University of Sheffield, Sheffield, UK; ^3^The Department of Community Medicine, University of Jos, Jos, Nigeria; ^4^Medical Research Council Unit, The Gambia at the London School of Hygiene and Tropical Medicine, Faraja, The Gambia; ^5^School of Allied and Public Health Professions, Canterbury Christ Church University, Canterbury, UK

 We would like to appreciate the interest of the four commentaries^1-4^ which were published in this journal in response to our paper^5^ and reflect on important aspects highlighted by the authors; biomedical hegemony and colonialism, institutional power, the hybridisation of biomedical power and new public management (NPM).

 We concur with Dalglish et al^4^ and Parashar et al,^1^ that *‘the dominance of medical professionals in healthcare is global, but it takes a particular shape in many low- and middle-income countries due to the imprint of colonialism.’*^2^ Johnson argues that an understanding of medical professional power in post-colonial countries can only be achieved by acknowledging the relationship between the medical professionals and their colonial and post-colonial states.^6^ In the case of Nigeria, the transmission of power, social status and authority of medical professionals has been through a historic colonial symbiotic relationship between the Imperial state and medical professionals,^7^ discoveries in tropical medicine,^8^ and more recently (demonstrated in our paper) biomedical epistemic communities.^5^ While our paper did not investigate some of these colonial origins in detail, we highlighted the trends that continue to “*situate doctors at the ‘top’ of a biomedical hierarchy and that have traditionally situated biomedicine above public health, traditional systems of medicine and other approaches to health*.”^1^ However, (biomedical) power is diffuse and not concentrated; this compels us to conceptualise it within a broader perspective by analysing areas in society where this power is reproduced and how it structures society. Therefore, to explore biomedical power, analysis must go beyond the domains of the medical profession,^4^ by analysing biomedical power and its discourse which are tied to specific institutions and actors such as the Global Fund.

 In the global context, the biomedical paradigm has dominated global health institutions such as the World Health Organization (WHO),^9^ and this paradigm is established in similar institutions, thus shaping the choices of both global and local actors.^10^ This is captured in Kapilashrami’s response which acknowledges our argument that the health policy process is a result of the reproductive nature of the biomedical discourse and its structural power over global and local health institutions.^3^ Kapilashrami’s response highlights why researchers need to focus not only on the biomedical dominance and medical professional power capture of local policy spaces, but a more nuanced exploration of ‘*the hegemonic structures (systems and protocols) and discourse (ideas and meanings) in constituting and constructing practices that legitimise, give meaning and stabilise the fund and health system governance*.’^3^ The institutionalisation of the biomedical discourse in global health institutions has shaped the direction in which decisions are taken in agenda setting, limiting the options available in creating solutions to problems. This biomedical institutionalisation of global health structures has been attributed to epistemic networks that dominate the global health policy spaces.^9^ In other words, institutional power may also be understood as certain actors exercising both structural and productive power through institutions in order to exercise indirect control over others.^10^ *‘Institutional power is actors’ control of others in indirect ways…through the rules and procedures that define those institutions, guides, steers, and constrains the actions (or non-actions) and conditions of existence of others.’*^10^ For example, NPM in combination with the biomedical discourse, is one of the tools used in exerting control over actors in health institutions.

 It has been argued that NPM is one of the key components that developing countries need to use effectively in implementing health reforms to deliver equitable healthcare within limited resources.^11^ The Structural Adjustment Programme of the World Bank and International Monetary Fund was one of the first encounters of developing countries with NPM in healthcare reforms.^12^ More recently, the encounter of NPM with developing countries has been through aid agencies and donors, facilitated through epistemic knowledge networks.^12^ The massive increase in international monetary donations by private-public partnerships,^12^ has advanced the use of a more open competitive and market-oriented approach for better cost-effective use of these resources.^13^ In achieving perceived efficiency from health providers, an adoption of performance-based funding, incentive structures, market driven research and vertical approaches has consequently shaped the health market.^2^ Brown and Rhodes in their response brilliantly summarised this point as follows *‘Recognizing this helps to explain the donor preference for earmarked funding and vertical programs as well as the bias often given to supply-side clinical and biomedical projects.*’^2^ However,a review of the empirical evidence concerning ‘pay for performance’ incentives by Global Health Initiatives, shows that incentives can have negative effects on the professionalism of health workers in general, leading to a *‘focus only on achieving the explicit targets that are being rewarded at the expense of other important but unmeasured tasks.’*^14^ This dynamic has limited the opportunities for community-led participation ‘*ultimately side-lining local expertise and community perspectives.*’^2^ If this dynamic is left unchecked, we could risk reducing the very idea and values of public health to commodified NPM driven objectives in the form of artificial intelligence (AI) algorithms, consequently, side-lining the community.

 Finally, due to the complexity of the various dimensions of power, researchers need to link the forms of power by answering questions that explain how discourse (productive power) create networks (structural power) and in turn, how these networks influence institutions (institutional power).^4^ Even though the biomedical narrative favours certain elite medical professionals, the hybrid of NPM and biomedicine poses potential threats to the professional power of frontline medical professionals through the process of ‘deprofessionalisation’^15^ and the subsequent brain drain of frontline medical professionals in low- and middle-income countries. By exploring concurrently professionalisation (professional monopoly) and deprofessionalisation (declining professional monopoly) in the forms of AI and NPM health management tools such as ‘task-shifting,’ we are able to observe the two processes in action that can expose the intersectionality in professional boundary disputes,^1^ dominance of biomedical cadres,^4^ and market dynamics in global health financing.^2^

## Ethical issues

 The original study had ethical approval obtained from the National Health Research Ethics Committee, Nigeria of the Ministry of Health in Nigeria through the University of Jos and the School of Health and Health Related Research (ScHARR) Research Ethics Committee at the University of Sheffield, UK. The CCM in Nigeria also gave approval and consent for their members to be recruited for interviews. Individual written informed consent was obtained from each participant prior to data collection.

## Competing interests

 Authors declare that they have no competing interests.
